# Templated replication (or lack thereof) under prebiotically pertinent conditions

**DOI:** 10.1038/s41598-018-33157-9

**Published:** 2018-10-09

**Authors:** Niraja V. Bapat, Sudha Rajamani

**Affiliations:** Indian Institute of Science Education and Research (IISER), Dr. Homi Bhabha Road, Pashan, Pune, 411 008 Maharashtra India

## Abstract

Accurate replication of encoded information would have been crucial for the formation and propagation of functional ribozymes during the early evolution of life. Studies aimed at understanding prebiotically pertinent nonenzymatic reactions have predominantly used activated nucleotides. However, the existence of concentrated pools of activated monomers on prebiotic Earth is debatable. In this study, we explored the feasibility of nonenzymatic copying reactions using the more prebiotically relevant 5′-nucleoside monophosphates (5′-NMP). These reactions, involving a 20-mer primer, were performed in the presence of amphiphiles, under volcanic geothermal conditions. Interestingly, the extended primer was not comparable to the expected full length 21-mer product. Our results suggest loss of the nitrogenous base in the extended primer. This phenomenon persisted even after lowering the temperature and when different rehydration solutions were used. We envisage that the loss of the informational moiety on the incoming 5′-NMP, might be occurring during addition of this monomer to the pre-existing oligomer. Significantly, when 5′-ribose monophosphate was used, multiple additions to the aforementioned primer were observed that resulted in hybrid polymers. Such hybrid oligomers could have been important for exploring a vast chemical space of plausible alternate nucleobases, thus having important implications for the origin of primitive informational polymers.

## Introduction

A widely accepted hypothesis pertaining to the existence of a putative ‘RNA World’ presumes that RNA molecules played a central role during the emergence and evolution of early life on Earth. Central to this hypothesis is the notion of an ‘RNA replicase’ enzyme (a ribozyme) that is capable of total self-replication^1^. However, prior to the advent of ribozymes that could catalyze reactions efficiently, it is thought that enzyme-free propagation of genetic information would have been a fundamentally crucial step. Inspired by this line of thought, studies have been undertaken in the last few decades to characterize nonenzymatic polymerization and information transfer reactions of nucleic acids. Most of these aforementioned studies have involved the use of activated nucleotides, and in some cases chemically modified primers, in order to facilitate the formation of either phosphodiester or phosphoramidite bond and, to also, obtain products in detectable yields. Several different activation chemistries, including imidazole, 2-methylimidazole, 1-methyladenine, 2-aminoimidazole^2,3^, oxyazabenzotriazole^4^ etc., have been explored thus far to successfully demonstrate enzyme-free oligomerization and copying reactions. Although a recent finding demonstrates a plausible route for the synthesis of imidazole activated ribonucleotides^5^, the presence or existence of significant amounts of most of the aforementioned activated nucleotides on the prebiotic Earth, is still arguable. On the other hand, possible prebiotic routes for the syntheses of nucleotides, and of their precursors, have been demonstrated in previous studies^2,6–8^. Very few reports, however, have demonstrated and characterized the products resulting from the oligomerization of non-activated monomers such as nucleoside-5′- monophosphates^9–11^. Importantly, the kinetics and fidelity of enzyme-free template-directed primer extension reactions of oligomers, in a putative RNA World, using non-activated nucleotides, still remains largely unexplored. Therefore, it becomes pertinent and important to study the feasibility of nonenzymatic information transfer and related processes, using non-activated nucleotides, which are prebiotically relevant in comparison to their activated counterparts.

Phosphodiester bond formation between an existing nucleic acid polymer and an incoming 5′-NMP is a slow reaction due to lack of a good leaving group such as an imidazole moiety on the phosphate. Furthermore, polymerization is essentially a condensation reaction involving the loss of a water molecule. Therefore, the rate of this reaction cannot be enhanced in the presence of bulk water. Not surprisingly, alternating cycles of dehydration and rehydration (DH-RH cycles) at elevated temperatures, have been shown to promote the polymerization of nucleic acid monomers^12^ as well as alpha hydroxy acids^13^ and malic acid monomers^14^. Dehydration at high temperatures not only results in concentration of the reactants, it also enhances the loss of water molecules, facilitating condensation and, thus, promoting bond formation. Such alternate DH-RH cycles would have been facilitated on the prebiotic Earth by diurnal cycles, seasonal variations etc. For e.g., dehydration in relevant niches might have been facilitated by high temperatures, which is followed by rehydration that results due to condensation of water at lower temperatures. It is thought that such alternate DH-RH cycles would have been abundant in terrestrial geothermal fields and in inter-tidal pools, geological features that are thought to have been prevalent on the early Earth. In a related study, researchers studied the copying of information from a single-stranded DNA, using deoxyribonucleotide monomers, in the presence of lipids, under DH-RH conditions^15^. Lipids are known to form liquid-crystalline matrices under dehydrated conditions, facilitating the concentration of the starting reactants (e.g. nucleotides) within the inter-layers of the resultant multilamellar structures^16,17^. This has previously been shown to facilitate the polymerization of 5′-NMPs, under DH-RH conditions and in the presence of phospholipids^9,11^. However, to our knowledge, no study has been reported to date wherein information transfer using RNA, and prebiotically relevant non-activated nucleotides, has been systematically evaluated especially under prebiotically relevant volcanic geothermal conditions. These RNA based studies are crucial as they pertain to a molecule whose relevance to the emergence of early life has been demonstrated in multiple lines of studies.

In this particular study, we aimed to discern phospholipid-assisted template-directed primer extension reactions, under DH-RH regimes and at elevated temperatures. As indicated earlier, this is likely the first study where in template-directed information transfer from RNA has been attempted using non-activated 5′-NMP nucleotides. As in previous comparable studies, we used the 3′-amino-2′, 3′-dideoxynucleotide terminated primer (Amino-G primer), to study pertinent reactions, as it will enable the detection of the extended product, which is known to form in observable yields^18–20^. The effects of various parameters on these reactions have also been systematically characterized. Importantly, we also report the extension of a pre-existing RNA primer using 5′-ribose monophosphate (5′-rMP), a sugar-phosphate monomer, that resulted in hybrid polymers. Such hybrid polymers could have potentially been significant in sampling different bases during the emergence of primitive functional polymers on the early Earth^11^.

## Results

Previously, few studies reported the polymerization of 5′-NMPs under DH-RH regimens, at low pH and in a range of high temperatures^9,11^. Furthermore, the presence of lipid molecules in the starting reaction mixture was shown to enhance the polymerization process. This is because lipids are known to form multilamellar structures under dehydrated conditions, allowing for the entrapment of the nucleotide monomers, thus facilitating their polymerization. Therefore, we set out to study whether template-directed extension of an existing RNA primer was also possible under similar conditions, using non-activated nucleotides.

### Extension of RNA primer under DH-RH regimen

**Figure 1:**
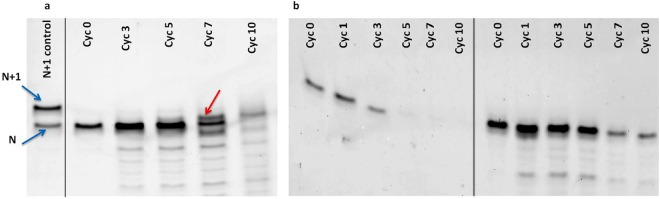
(**a**) Extension of the RNA primer using MisInc_U template and 5′-AMP as the monomer, in the presence of lipid, over repeated cycles of DH-RH. The reactions were carried out at 90 °C with 1 mM H_2_SO_4_ as the rehydrating agent. The red arrow indicates the extended product. In the control lane, ‘N’ indicates the 20-mer RNA primer while ‘N + 1’ indicates extension of the primer by one nucleotide^20^. (**b**) Stability of the RNA primer over multiple DH-RH cycles, when performed in the absence of lipid (left panel) and in the presence of lipid (right panel) in the starting reaction mixture. The black vertical lines in the above two panels have been used to demarcate two reaction sets that were run on the same gel (Panel b). In Panel a, the black line demarcates a reaction set from the control lane, both of which were analysed on the same gel.

In preliminary reactions, 1, 2-dilauroyl-sn-glycero-3-phosphocholine (DLPC) lipid vesicles and 5′- NMPs were added to a final concentration of 5 mM and 10 mM respectively, to the pre-annealed RNA primer-template complex. This mixture was subjected to repeated cycles of DH-RH at 90 °C, and 1 mM H_2_SO_4_ was used as rehydration solution in these reactions to facilitate an acid-catalyzed esterification type reaction that has been demonstrated previously^14,15^. Upon analysis using denaturing gel electrophoresis, a band for the extended primer product was observed over increasing DH-RH cycles. However, this new band was not comparable to the expected 21-mer product (i.e. one nucleotide addition to the original 20-mer primer). In fact, the newly formed band did not run to the same extent as an intact 21-mer band would have, on the 20% denaturing urea gel that was used for analyzing the progress of the reaction (first lane of Fig. 1a, intact 21-mer band indicated by a blue arrow). The band obtained was actually found to be somewhere in between that of a 20-mer and 21-mer RNA (indicated by a red arrow in ‘Cyc 10’ lane of Fig. 1a). This was true for all of the 5′-NMP-based reactions (N = A/U/G/C), irrespective of which one was used as the starting monomer in a given reaction. We suspect that the extended product in these reactions might have an abasic site, a result that has previously been reported for comparable concatenation reactions that were carried out under similar conditions^11^. Furthermore, we observed higher degradation of the fluorescently-labelled RNA primer over multiple cycles of DH-RH, when similar reactions were carried out in the absence of lipid (Fig. 1b left panel vs right panel). Comparable results were also observed when a longer chain-length phospholipid, namely 1-palmitoyl-2-oleoyl-*sn*-glycero-3-phosphocholine (POPC), was used instead of DLPC, in the starting reaction mixture, suggesting a protective role for the lipids under these harsh prebiotically pertinent conditions. Additionally, in complementary studies, we also performed the aforementioned main experiment i.e. attempted templated-replication in the presence of amphiphiles, using a canonical hydroxyl terminated RNA primer (Hydroxyl-G primer). Since hydroxyl group is known to be less nucleophilic than an amino group, we did not see the formation of prominent amount of the extended primer product (Fig. S1).

### Effect of temperature and rehydrating solution on primer extension

**Figure 2:**
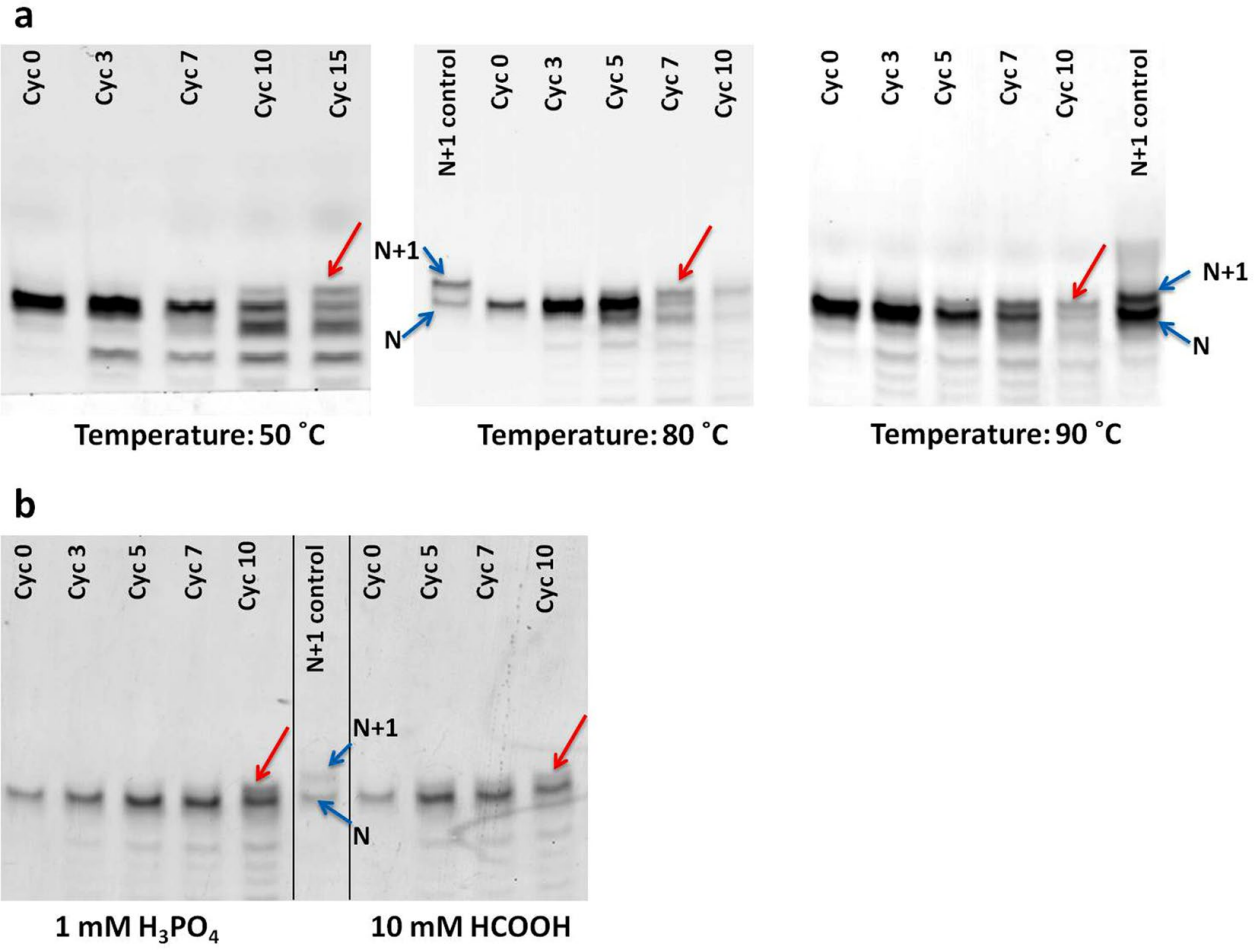
Extension of RNA primer in the presence of MisInc_U template, 5′-AMP as monomer and lipid (**a**) at different reaction temperatures (**b**) using different rehydrating agents. The red arrow indicates the extended primer product. The black vertical lines in the Panel b gel image have been used to demarcate reactions sets run on the same gel. Additionally, the black line has also been used to demarcate reactions from the control lane. ‘N’ indicates the 20-mer RNA primer and ‘N + 1’ indicates extension of the primer by one nucleotide.

Loss of the nucleotide base is known to occur under high temperature and low pH conditions as the glycosidic bonds are susceptible to hydrolysis in such conditions^21,22^. Given that the extension of the primer at 90 °C, under DH-RH conditions, resulted in a product with a possible abasic site, the efficiency of the extension reaction was tested at lower temperatures. All the reactions mentioned henceforth were carried out using Amino-G primer, and in the presence of lipids. Extension of the Amino-G primer was observed at lower temperatures, including at 80 °C and at 50 °C (Fig. 2a, middle and left panels respectively). It is to be noted that lowering of the temperature led to an increase in the dehydration time for each cycle. For e.g., the dehydration time at 50 °C increased to up to 90 minutes (from 30 minutes at 90 °C) due to slower evaporation rate. Importantly, the resulting extension product still ran lower than the intact 21-mer primer product that was used as control on the gel.

Furthermore, we also assessed the effect of different rehydrating agents on the potential loss of base during primer extension reactions, under DH-RH cycles at 90 °C. To begin with, different concentrations of H_2_SO_4_, varying from 0.05 mM to 5 mM, were used, amongst which 1 mM H_2_SO_4_ was found to be the optimum concentration. Higher concentrations of acid led to huge degradation of RNA during the cycling reactions whereas at lower concentrations of acid, no primer extension was observed (Fig. S2). Subsequently, different types of acids were also investigated as rehydrating solutions to check if any of them could prevent the possible loss of base during primer extension, under aforementioned conditions. Amongst the different mineral and organic acids that were tested, the extended primer product was detected when phosphoric acid and formic acid were used as rehydrating agents (Figs 2b and S3). However, the extended primer continued to run lower than an intact N + 1 (21-mer) product (bands indicated by red arrows in Fig. 2b).

**Figure 3:**
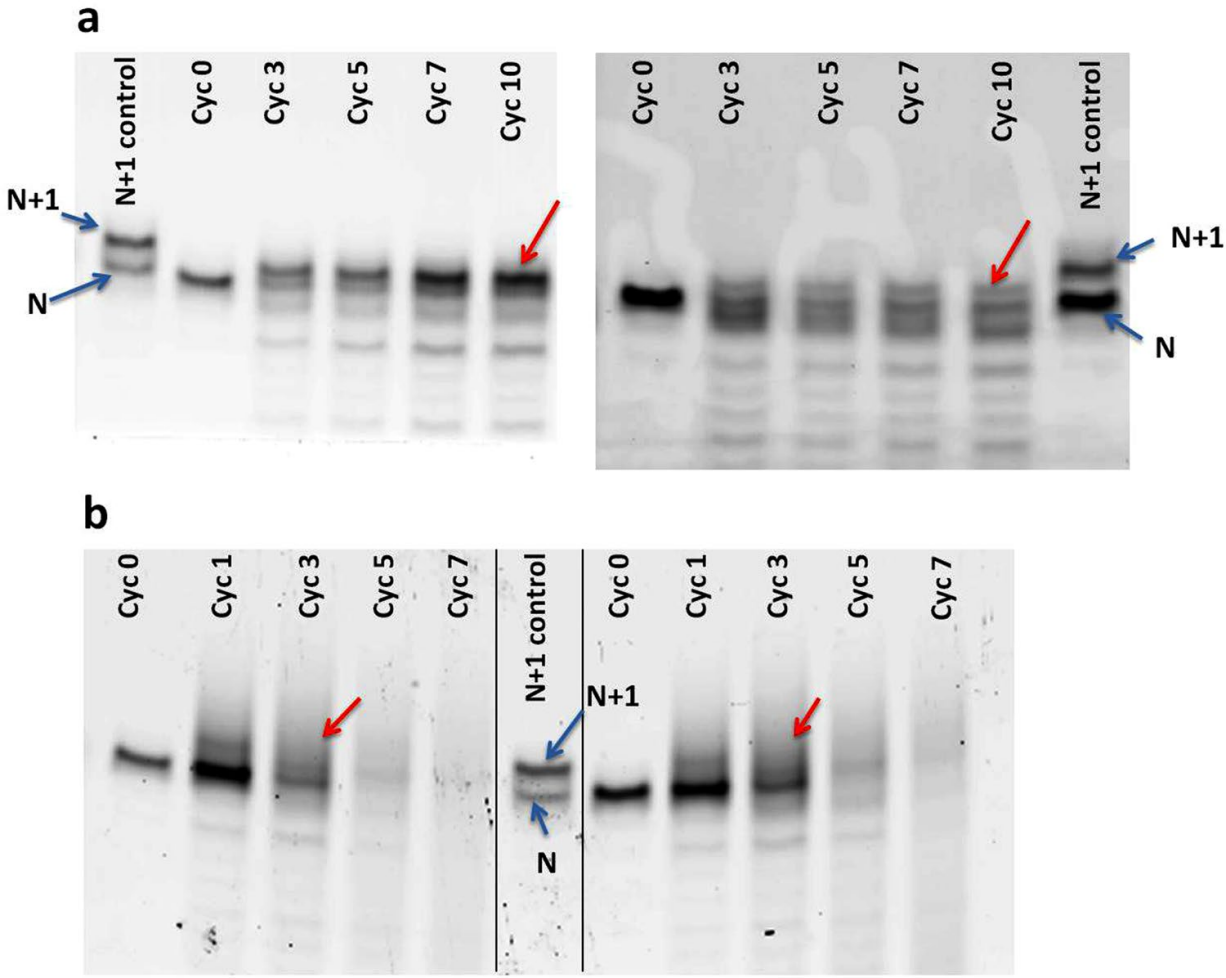
Extension of the RNA primer using (**a**) 1 mM 5′-AMP with 5 mM lipid (left panel) and 1 mM 5′-AMP with 1 mM lipid (right panel), in the starting reaction mixture. The reactions contained 1 mM H_2_SO_4_ and nanopure water was used as the rehydrating agent. (**b**) 10 mM of 5′-rMP, in the absence (left panel), and the presence (right panel) of the template in the reaction. Reactions were carried out at 90 °C, in the presence of 5 mM lipid and 1 mM H_2_SO_4_ was used as rehydrating agent. The black vertical lines in the Panel b gel image, demarcates two different reactions run on the same gel. Red arrows indicate extended RNA primer product/s. ‘N’ indicates the 20-mer RNA primer while ‘N + 1’ indicates extension of the primer by one nucleotide.

Consequently, the concentration of the acid in the reaction mixture was kept low to see if this might prevent what seemed like the generation of an abasic site. Towards this attempt, sulphuric acid was added to the starting reaction mixture to a final concentration of 1 mM (to maintain the requisite pH) and nanopure water was used as rehydrating agent for the remainder of the DH-RH cycles. Using this approach, the extended primer product was observed at a starting concentration of 5′-NMP that was as low as 1 mM, albeit at a lower efficiency (Table S1). Furthermore, the extension was also observed at a lower concentration of the lipid (as low as 1 mM) in the starting reaction mixture (indicated by a red arrow in Fig. 3a). However, even at this low concentration of acid in the reaction mixture, the extended primer product still did not run at a level similar to that of a known 21-mer RNA control.

### Effect of monovalent cations

A recent study from Da Silva *et al*. reported the formation of oligomers from 5′-NMPs in salty environments, and under cycling conditions at high temperatures^23^. Interestingly, the RNA-like oligomers formed in their reactions were shown to have base-pairing ability, as was evident by the ethidium bromide staining of the resultant products. Amongst the different salts that they had used, ammonium chloride was found to be very efficient in promoting the formation of oligomers. To analyze whether the presence of NH_4_^+^ ions might result in the formation of an intact extended primer product, NH_4_Cl was added, either in isolation or along with NaCl, to the reaction mixtures. The final concentration for each of the salt was maintained at 200 mM. However, the presence of NH_4_Cl did not improve the efficiency of our primer extension reactions in terms of resulting in an intact N + 1 extended primer. The resultant product band continued to migrate in between that of a known 20-mer and 21-mer RNA on the denaturing gel (Fig. S4). We also observed higher amounts of RNA degradation during the course of DH-RH reactions in the presence of ammonium ions, as opposed to when only sodium ions were present in the reaction (Compare Fig. S4 vs. Fig. 1a).

### RNA primer extension using 5′-ribose monophosphate

5′- ribose monophosphate (5′-rMP) is a monomer which contains a sugar and a phosphate entity but lacks any information carrying nitrogenous base (which is otherwise present in 5′-NMPs). Earlier studies indicate that 5′-rMP polymerizes readily under fluctuating DH-RH conditions, to potentially result in linear and cyclic sugar-phosphate backbones^11^. However, the addition of such a backbone onto a pre-existing RNA oligomer has not yet been evaluated. Towards this, primer extension reactions were carried out under DH-RH regimes at 90 °C, using 5′-rMP as monomers as against the 5′-NMPs (used in the earlier reactions). Denaturing gel analysis showed the presence of a long smear of products, even in as early as the end of the first DH-RH cycle. The smudge (as opposed to a clear single extension product band), observed on the top of the RNA primer band, is suggestive of possible multiple additions to the primer. Interestingly, this was seen, both, in the presence and absence of the RNA template (control reaction) in the starting reaction mixture (Fig. 3b right vs left panels). Similar results were also observed when using as low as 1 mM of 5′-rMP in the starting reaction mixture, along-with 1 mM sulphuric acid and nanopure water as rehydration agent (data not shown). In contrast, in a parallel reaction wherein only 5′-rMP was cycled under aforementioned conditions, no detectable fluorescent signal was obtained upon gel analysis. Significantly, the primer extension product from the addition of a single 5′-rMP monomer, and that obtained when using 5′-AMP as monomer (discussed in previous results), were found to be running at the same level upon gel analysis (Fig. S5). This provided an indirect confirmation of the possibility that in our main reactions involving 5′-NMPs, the addition of an abasic nucleotide was resulting in the primer extension band that did not run to the same length as an intact 21-mer product.

## Discussion

Chemically driven oligomerization and nonenzymatic template-directed reactions are uphill processes as the formation of phosphodiester bonds between the non-activated nucleotides is not a spontaneous reaction. In order to demonstrate polymer formation from non-activated nucleoside monophosphates, previous studies have reported the use of alternate DH-RH cycles at elevated temperatures, to facilitate the aforementioned processes^9–11,15^. The dehydration phase helps in concentrating the monomers, thus increasing the chances of bond formation between them^12^. Subsequent rehydration helps in the random re-distribution of, both, the monomers as well as the resultant oligomers. This increases the probability of formation of longer oligomers over multiple wet-dry cycles. Therefore, by trapping the polymers in a kinetic trap, wherein the rate of polymer formation exceeds the rate of polymer degradation, alternate DH-RH cycles ultimately help in yielding polymers from the starting monomers. The elevated temperature of the reaction decreases water activity, thus favouring the forward reaction by overcoming the energy barrier for the phosphodiester bond formation. Furthermore, the efficiency of these reactions has been found to be higher at low pH. A suggested mechanism, similar to that of acid-catalyzed esterification, hypothesizes the role of protonation of the nucleotide at lower pH, thus facilitating the formation and accumulation of oligomers over repeated DH-RH cycles^15^. Previous studies have also characterized the use of lipids in such reactions, which reduces the unfavourable back reaction and results in an apparent increase in the yield of oligomers^9^. Phospholipids are known to form multi-lamellar sandwiches of alternating hydrophilic and hydrophobic layers in the dehydrated phase of the reaction. Studies have shown the nucleotide monomers to get confined in the hydrophilic layers, thus increasing their chance of participating in a phosphodiester bond formation^16,17^. Significantly, during the subsequent rehydration phase, some of the oligomers that form in the dehydration phase could get encapsulated in the lipid vesicles, a step that is considered crucial for the emergence of protocells.

Faithful replication of any functional (catalytic) polymer that would have resulted from the aforementioned polymerization reactions under DH-RH scenarios, would have been a crucial step for them to have consistently acted as a catalyst. Only one study has been reported so far, where nonenzymatic replication reactions have been attempted under similar DH-RH reaction regimens. The authors of this study, however, used a mixture of 2′-deoxyribonucleoside 5′-monophspahtes and a single stranded DNA, as template, to look at nonenzymatic replication^15^. We, therefore, set out to test if the DH-RH conditions would also support template-directed primer extensions using RNA monomers, which is directly pertinent to the ‘RNA World hypothesis’, with imminent implications for the emergence of early life.

To begin with, the extension of a pre-existing RNA primer (Amino-G primer) was analyzed. The 3′- amino group, present at the primer’s extending terminus, acts as a better nucleophile than the contemporary hydroxyl group, thus yielding detectable amounts of the extended product (Fig. S1). Extension of the Amino-G primer was observed upon multiple cycles of rehydration and dehydration. The presence of lipids in the reaction mixture seemed to confer some protection under our experimental conditions. This might potentially result from sequestration of the RNA within the hydrophilic layers of the multilamellar structures that are formed during the dehydrated phase^16^. The newly formed product’s retention time on the denaturing gel, however, was found to be in between that of the unreacted primer (20-mer) and that of known 21-mer control RNA (Fig. 1a). This is indicative of the product possibly having an abasic site as the charge on the product seems to be more than that of the 20-mer primer from our gel analyses. Previous results have demonstrated the formation of RNA-like, depurinated oligomers from non-activated 5′-AMP, upon multiple DH-RH cycles^11^. Our results suggest that even template-directed primer extension reactions are potentially susceptible to base loss during bond formation. This seems to be more prevalent on the 3′-end of the growing polymer, because if the 5′-end of the RNA primer used in this study happened to be degraded, it cannot be visualized on the gel as the Cy3 fluorescent tag is attached to the 5′-end of the primer. To further confirm the aforementioned, we subjected the 21-mer intact primer to similar reaction conditions. We observed that the 21-mer primer degraded directly to a 20-mer oligomer (corresponding to the starting primer length), over multiple DH-RH cycles (Fig. S6). Additionally, no intermediate product was observed in between the bands corresponding to a 20-mer (starting primer) and 21-mer (extended product) length RNA. This strongly hints at the potential loss of a base on the incoming 5′-NMP ribonucleotide. This possibility could occur just before or during the addition of the incoming nucleotide, onto the pre-existing primer, during the course of our DH-RH reactions. During template-directed primer extension, the template is thought to stabilize the incoming base via near neighbour interactions. However, templating effect does not seem to be able to prevent the potential loss of base in our reactions, further suggesting that it could result from the chemical reaction that takes place during the actual bond formation between the incoming nucleotide and the primer. Additionally, the temperature at which the DH-RH cycles were carried out was reduced systematically to check for any effect on this phenomenon. However, the apparent loss of the informational entity seemed to persist even at temperatures as low as 50 °C. Therefore, it seems that the low pH of the reaction might possibly be the predominant reason for this effect in our reactions.

We also tried different rehydrating agents other than sulphuric acid to study their effect on template-directed primer extension reactions using 5′-NMPs. In total, four mineral acids (viz. H_2_SO_4_, HCl, HNO_3_, and H_3_PO_4_) and two organic acids (viz. formic acid and acetic acid) were evaluated. Typically 1 mM of mineral acid was used while up to 10 mM of organic acids had to be used to compensate for their lower intrinsic strength. Among the mineral acids used, we observed the extension of the primer only when either sulphuric acid or phosphoric acid was used as the rehydration agent. This might potentially be due to the thermally labile nature of the other mineral acids that we tried, as the reactions were carried out at 90 °C. Among the organic acids that were used, we observed primer extension only when formic acid was used. This might be attributed to the fact that formic acid is a stronger acid than acetic acid, and hence might be more efficient in bringing down the pH of the reaction. Significantly, the extended product’s retention time on the gel continued to not correspond to that of the expected intact N + 1 nucleotide extension. Furthermore, we checked whether addition of ammonium cations to our reaction might facilitate efficient template-directed primer extension as was observed in a previous study^23^. However the presence of NH_4_^+^, either alone or along with Na^+^ ions, did not result in an apparent increase in the efficiency of the extended product formation (Fig. S4). Furthermore, the presence of ammonium ions in the reactions mixture, also lead to greater RNA degradation under our DH-RH reaction regimen.

Finally, to analyze whether the repeated addition of sulphuric acid after every DH-RH cycle, was causing what seemed like an apparent base loss, due to higher concentrations of acid in the reaction, nanopure water was used instead as the rehydrating agent. In these reactions, sulphuric acid was added to the starting mix to reduce the pH to around 3.5 and was kept at a constant 1 mM throughout the course of the DH-RH cycling. Even upon reduction in the amount of acid in the reaction mixture, the extended product continued to run lower than the expected intact N + 1 primer product (Fig. 3a). Interestingly, the extension of the RNA primer continued to occur even while using as low as 1 mM of the monomer. We believe this result is significant, as the existence of highly concentrated pools of nucleotides on the prebiotic Earth is not considered very likely.

Despite varying several parameters of the reaction scheme, including pH, temperature, nature and strength of the rehydration solution, and that of the monovalent cations, the extended primer product had a retention time on the gel that was lower than that of a known 21-mer control. This indicated a strong probability of a base loss over the reaction course and it seemed that the primer was getting extended by the addition of only a sugar-phophate moiety. Given this possibility, we also analysed whether ribose-5′-monophosphate (5′-rMP) by itself could extend the pre-existing RNA primer. Oligomer formation from 5′-rMP monomers, under alternate DH-RH conditions, has been previously reported^11^. Upon cycling the RNA primer with 5′-rMP, long streaks were observed on the denaturing gel. These resultant products were observed even at the end of the first DH-RH cycle itself (Fig. 3b ‘Cyc 1’ lane). This seemed to indicate a higher polymerization potential for the rMP monomers in comparison to contemporary ribonucleotides. Also, the presence of long streaks on the denaturing gel indicated possible multiple additions of rMP to the RNA primer. Both linear and cyclic oligomers of rMP have been previously reported to result upon multiple DH-RH cycles^11^. We suspect that the RNA primer is possibly getting extended by a mixture of, both, linear and cyclic rMP molecules, and this, in turn, resulted in poor resolution of these polymers on the denaturing gel. Furthermore, as a control, 5′- rMP molecules were cycled alone (in the absence of the primer and template) and analysed using gel electrophoresis. No streaks were observed upon fluorescence imaging of the gel, and even upon SyBrGold staining (due to lack of bases), confirming the possibility that the rMP oligomers were most likely covalently linked with the RNA primer in the test reactions involving 5′-rMP. This is significant as such hybrid molecules are hypothesized to have been important for exploring a vast chemical space of plausible alternate nucleobases that might have resulted in the formation of primitive information polymers of a pre-RNA World^24^. This hypothesis has gained support from recent studies that have demonstrated the addition of non-contemporary bases to ribose or ribose monophosphate, at both ambient conditions^25^ and at high temperature regimes^26,27^.

In conclusion, this study highlights the importance of systematically characterizing prebiotically pertinent reactions in simulated laboratory conditions to better understand how they might advent in niches that are thought to have supported the origin of life on prebiotic Earth. These findings particularly have important implications for discerning relevant mechanisms in such niches that would have eventually enabled the transition from chemistry to biology on Earth. Furthermore, the formation of hybrid polymers in our 5′-rMP based reactions, strengthens the likelihood of pre-RNA World hypothesis. Also, the possibility that the same sugar phosphate backbone can potentially sample both contemporary as well as alternate nucleobases, as discussed above, is indicative of the important role such mixed backbones might have played in the transition from a putative pre-RNA World to an RNA World.

## Material and Methods

### Materials

The disodium salts of all four 5′-NMPs viz. Adenosine 5′-monophosphate (AMP), Guanosine 5′-monophosphate (GMP), Uridine 5′-monophosphate (UMP), Cytidine 5′-monophosphate (CMP), and ribose 5′-monophosphate (5′-rMP) were purchased from Sigma-Aldrich and used without further purification. The RNA primers used in this study are Amino-G (acquired from Keck laboratory, Yale, USA) and Hydroxyl-G (acquired from Thermo Fisher Scientific, USA) primers. Both these primers had fluorescence labels on their 5′-end for facilitating their detection on polyacrylamide gel electrophoresis (PAGE). The Amino-G primer terminates with a 3′-amino-2′, 3′-dideoxynucleotide (Metkinen, Finland) while the Hydroxyl-G primer terminates in a canonical ribonucleotide. The sequences of the primers and templates are as given below, with the template base indicated in bold:

Primer Amino-G: 5′ GG GAU UAA UAC GAC UCA CUG-NH_2_.

Primer Hydroxyl-G: 5′ GG GAU UAA UAC GAC UCA CUG.

Template MisInc_C: 5′ AGU GAU CU**C** CAG UGA GUC GUA UUA AUC CC.

Template MisInc_G: 5′ AGU GAU CU**G** CAG UGA GUC GUA UUA AUC CC.

Template MisInc_A: 5′ AGU GAU CU**A** CAG UGA GUC GUA UUA AUC CC.

Template MisInc_U: 5′ AGU GAU CU**U** CAG UGA GUC GUA UUA AUC CC.

The phospholipid used in this study, namely 1, 2-dilauroyl-sn-glycero-3-phosphocholine (DLPC) was purchased from Avanti Polar Lipids Inc. All other reagents used were of analytical grade purchased from Sigma-Aldrich.

### Methods

#### Reaction setup

A typical reaction was set up by annealing 0.65 μM of primer and 1.3 μM of template to each other by heating at 95 °C for 5 minutes, followed by cooling at room temperature (RT). 100 mM Tris (pH 7.0) and 200 mM NaCl were then added to the annealed primer-template complex. A suspension of DLPC vesicles was prepared in nanopure water by hydrating the dried film of lipid, followed by extrusion of the vesicles through a 100 nm membrane using a mini extruder (Avanti Polar lipids Inc). DLPC vesicles and 5′-NMPs were added to the reaction mixture at a final concentration of 5 mM and 10 mM, unless otherwise specified. The reaction mixture was then allowed to dry at elevated temperature. The dried mixture was typically rehydrated after 30 minutes. After allowing the rehydration for 5 minutes, the procedure was repeated for several DH-RH cycles. A sample for analysis was removed in the rehydration phases at regular intervals.

#### Analysis of reaction products

The samples were collected in TBE buffer containing 8 M urea and 100 mM EDTA. The sample volumes were adjusted to a standardized amount to compensate for the degradation of RNA primer that occurs over multiple cycles of DH-RH. A competitor RNA, without any fluorescence tag and with a sequence that is exactly similar to that of the tagged primer, was used (5′ GG GAU UAA UAC GAC UCA CUG). This was added in at least 10 times excess to the reaction samples, to successfully separate the fluorescent primer in question for unhindered gel analysis. The extended primer products were analyzed on 20% denaturing PAGE and the gels were imaged with a Typhoon Trio plus imager (GE Healthcare) at 550PMT and 100 micron resolution setting, using the 532 nm excitation laser. All the gels were scanned by setting the region of interest (ROI) in the image acquisition software to the lower half of the gel region in the 17 × 15 cm gel, which typically has all bands pertinent to our reaction. This allowed for the efficient detection of the fluorescently labelled primer and the resultant extended products. The gel images were subsequently processed using ImageQuant v5.2 software to minimally adjust the contrast and also for quantification purposes in some cases.

## Electronic supplementary material

Supplementary Information

## Data Availability

All data generated or analysed during this study are included in this published article (and its Supplementary Information files).
