# Supplementary material for: Templated replication (or lack thereof) under prebiotically pertinent conditions
Source: Sci Rep. 2018 Oct 9;8:15032. doi: 10.1038/s41598-018-33157-9 (PMC6177409; doi:10.1038/s41598-018-33157-9)
Supplement: Supplementary file 1 — Supplementary Information [file 41598_2018_33157_MOESM1_ESM.pdf]

Templated replication (or lack thereof) under prebiotically pertinent conditions

Niraja V. Bapat, Sudha Rajamani*

Indian Institute of Science Education and Research (IISER), Dr. Homi Bhabha Road, Pashan,
Pune 4110 008, Maharashtra, India
* E-mail of Correspondence Author: srajamani@iiserpune.ac.in
(Telephone no: +91-020-25908061 Fax no: +91-020-25899790)

Supplementary Information

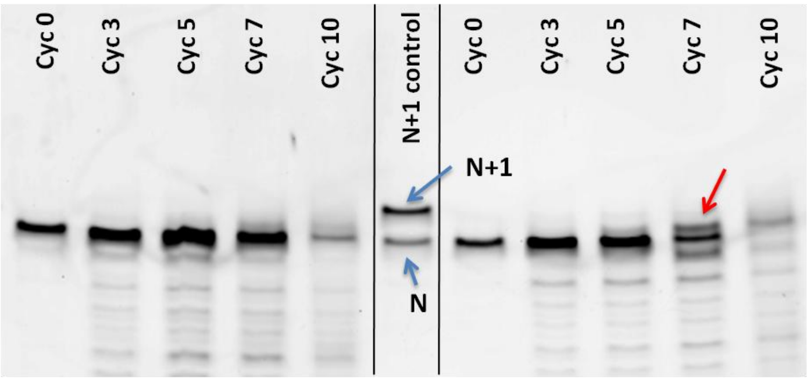

Figure S1: Extension of RNA primer ending with a canonical ribonucleotide (left panel) and a 3'-amino-2', 3'-dideoxynucleotide (right panel) over multiple cycles of DH-RH at 90˚C, in the presence of MisInc_U template, 5'-AMP (monomer) and lipid, using 1mM H_2SO_4 as rehydrating agent. The red arrow indicates the extended primer product, which is more apparent from Cyc 7 in the image on the right panel, as against the image on the left panel. 'N' indicates the 20-mer RNA primer, 'N+1' indicates extension of the primer by one nucleotide. The black lines on the gel images are used to demarcate different reactions and control lanes (which were run on the same gel) from each other.

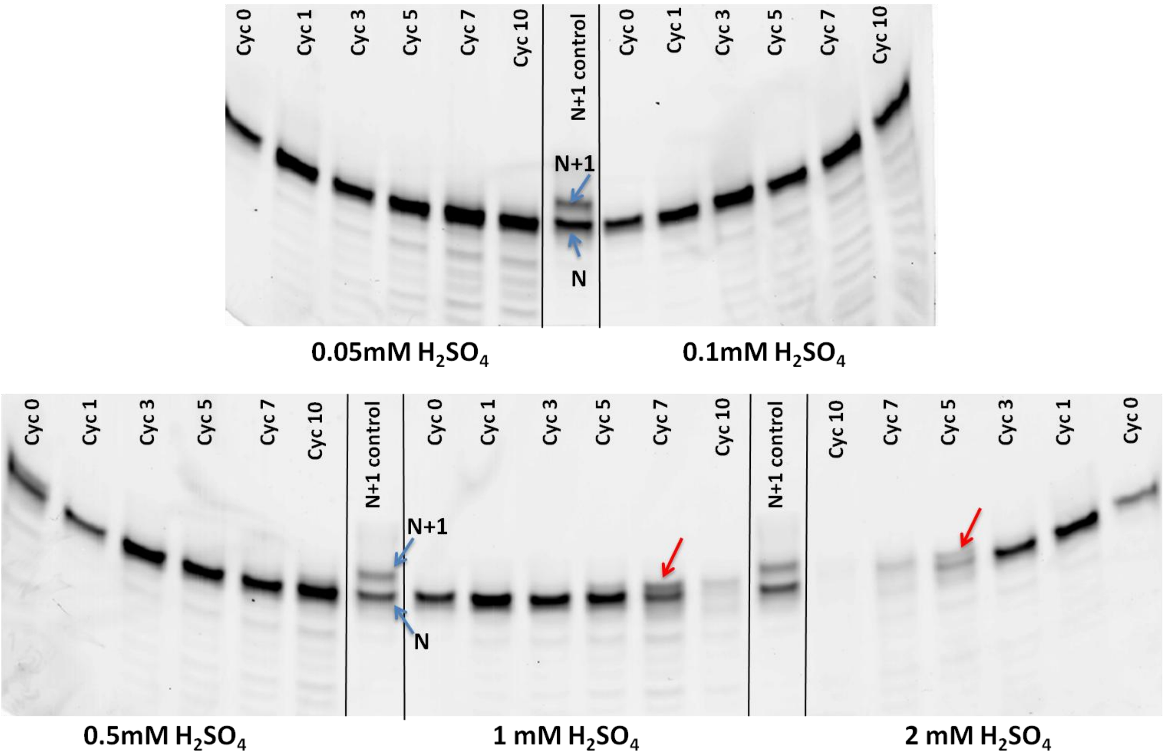

Figure S2: Extension of RNA primer over multiple cycles of DH-RH at 90˚C, in the presence of MisInc_U template, 5'-AMP (monomer) and lipid, using different concentrations of sulphuric acid as the rehydrating agent. The red arrow indicates the extended primer product. 'N' indicates the 20-mer RNA primer while 'N+1' indicates extension of the primer by one nucleotide. The black vertical lines on the gel images are used to demarcate different reactions and control lanes (which were run on the same gel), from each other. Note: The lanes, in the panel depicting the results where 2mM sulphuric acid was used as the rehydrating agent, is in descending order of cycle number.

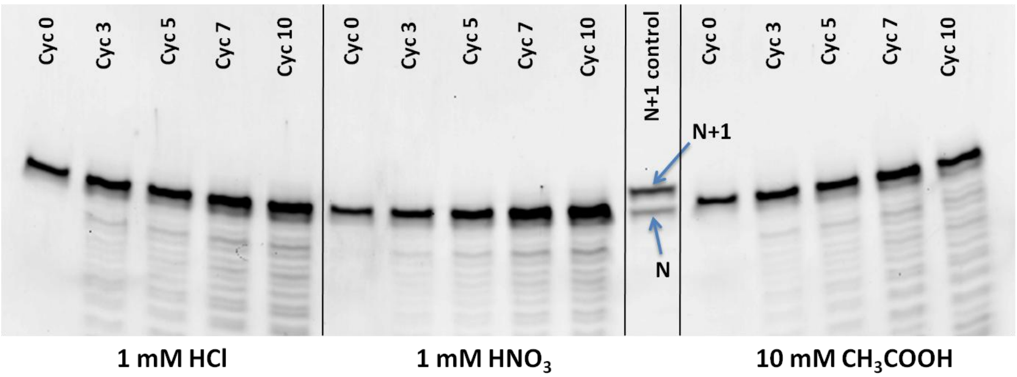

Figure S3: Experiment to check the extension of RNA primer at 90˚C, in the presence of MisInc_U template, 5'-AMP (monomer) and lipid, using different acids as the rehydrating agents. No extension product was observed in reactions wherein HCl, HNO_3 and CH_3COOH, respectively, were used as the rehydrating agent. 'N' indicates the 20-mer RNA primer, 'N+1' indicates extension of the primer by one nucleotide. The black vertical lines have been used to demarcate different reactions and control lanes (which were run on the same gel), from each other.

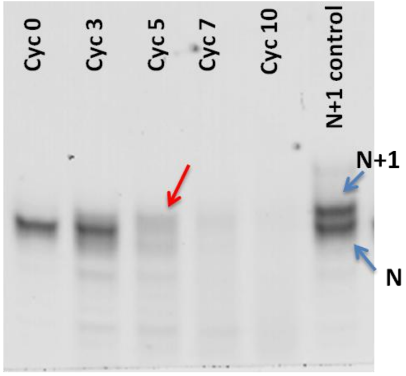

Figure S4: Extension of RNA primer, over multiple cycles of DH-RH, in the presence of MisInc_U template, 5'-AMP (monomer), lipid, and 200mM NH_4Cl. The red arrow indicates the extended primer product, which is faint in this image. The presence of ammonium ions did not seem to prevent the loss of base on the extended primer product. The reaction was carried out at 90˚C, using 1mM H_2SO_4 as rehydrating agent. As in previous images, 'N' indicates the 20-mer RNA primer while 'N+1' indicates extension of the primer by one nucleotide.

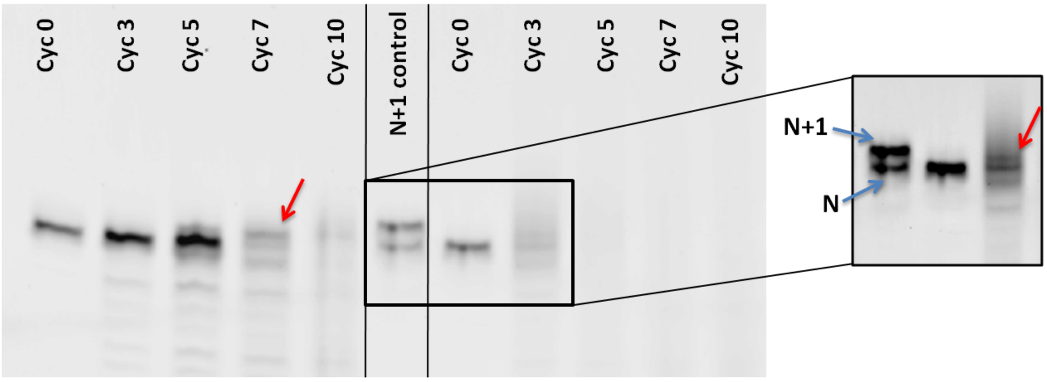

Figure S5: Extension of RNA primer over multiple cycles of DH-RH, in the presence of MisInc_U template, lipid, and 5'-AMP as monomer (left panel) or 5'-rMP as monomer (right panel). The inset shows the selected (boxed) part of the gel with the contrast enhanced for clearer visualization. The red arrow indicates the extended primer product. The extended primer products from both the reactions were observed to be running at the same level. The reactions were carried out at 90˚C, using 1mM H_2SO_4 as rehydrating agent. 'N' indicates the 20-mer RNA primer and 'N+1' indicates extension of the primer by one nucleotide. The black vertical lines on the gel images have been used to demarcate different reactions and control lanes (which were run on the same gel), from each other.

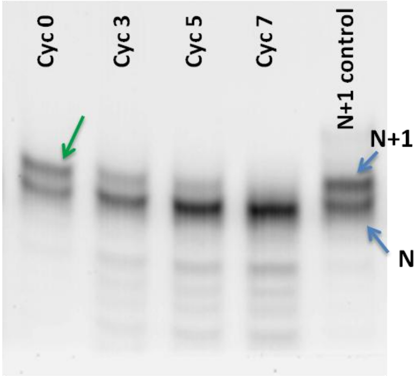

Figure S6: Degradation of the intact 21-mer primer (indicated by green arrow) over multiple cycles of DH-RH, in the presence of lipid. The reaction was carried out at 90˚C, using 1mM H_2SO_4 as rehydrating agent. The intact 21-mer primer product was obtained using MisInc_U template and adenosine-5'- phosphoimidazolide (ImpA) as monomers (1). The 21-mer primer breaks down to the 20-mer primer over time. Importantly, there was no intermediate band observed between the N and N+1 band in this reaction which was, otherwise, seen in all the primer extension reactions that involved 5'-NMPs under similar reaction regimen. 'N' indicates the 20-mer RNA primer and 'N+1' indicates extension of the primer by one nucleotide.

Table S1: Semi-quantitative yields of the extended primer product over multiple DH-RH cycles

Concentration of 5'-AMP	Rehydrating solution	Yield of extended primer product after 7 DH-RH cycles	Yield of extended primer product after 10 DH-RH cycles
10mM	1mM H_2SO_4	8.5%± 1.6%	2.4%± 0.2%
1mM	Nanopure water	1.8%± 0.07%	1.4%± 0.07%

The reactions were carried out at 90˚C, using MisInc_U template, 5’-AMP as the nucleotide monomer, in the presence of 5mM lipid. The starting monomer concentration and the rehydrating agent were varied in order to vary the amount of acid in the reaction mixture. Yields are calculated with respect to the intact 20-mer primer band intensity (at cycle 0) from two independent experiments and are represented as mean ± S.D. The reaction mixture with 1mM H_2SO_4 as rehydration agent had 60nmoles of H_2SO_4 at the end of 7th DH-RH cycle and 72nmoles of H_2SO_4 at the end of 10th DH-RH cycle. While, the reaction mixture with nanopure water as rehydration agent contained 10 nmoles of H_2SO_4 throughout all the DH-RH cycles.

Figure S7: The un-cropped original gel images for all the figures in main text of the manuscript and in supplementary information. The images were acquired using Typhoon Trio plus imager (GE Health Care) using the 532nm laser at 550PMT exposure and 100 micron resolution. The green box denotes the region of a particular gel that has been cropped and used in the figures of either the main text or in the supplementary information section (as indicated below). The (original) gels that were used without any cropping of the lanes (do not contain the green boxes) have also been included below.

Un-cropped original images for pertinent figures from the main text of the manuscript:

1. **Figure 1a**

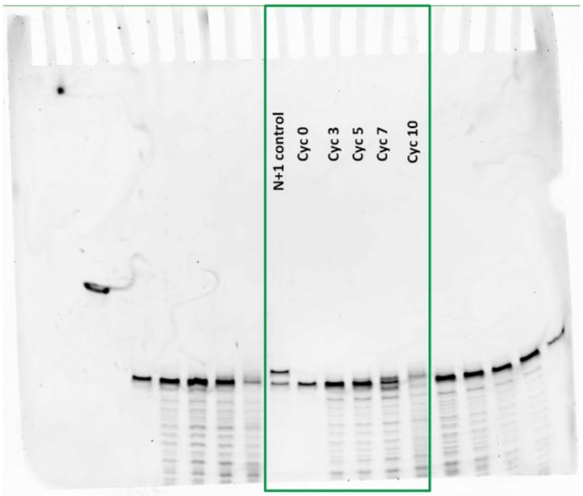

Extension of Amino G primer using MisInc_U template and 5'-AMP

2. **Figure 1b**

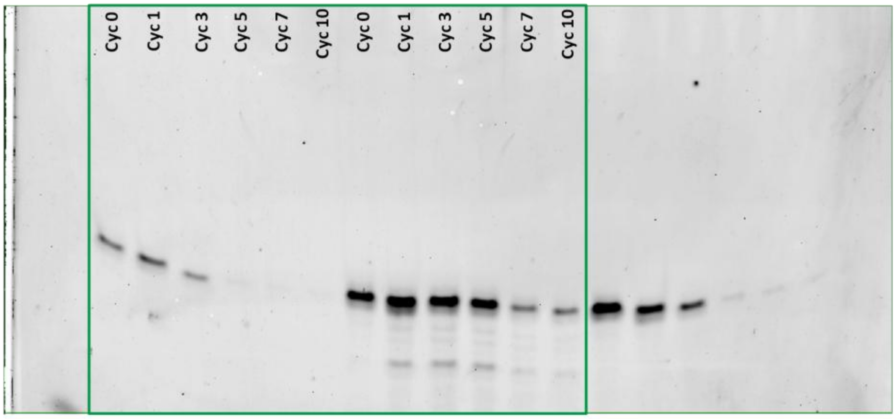

Stability of RNA primer in absence of lipid

Stability of RNA primer in presence of lipid

3. Figure 2a left panel

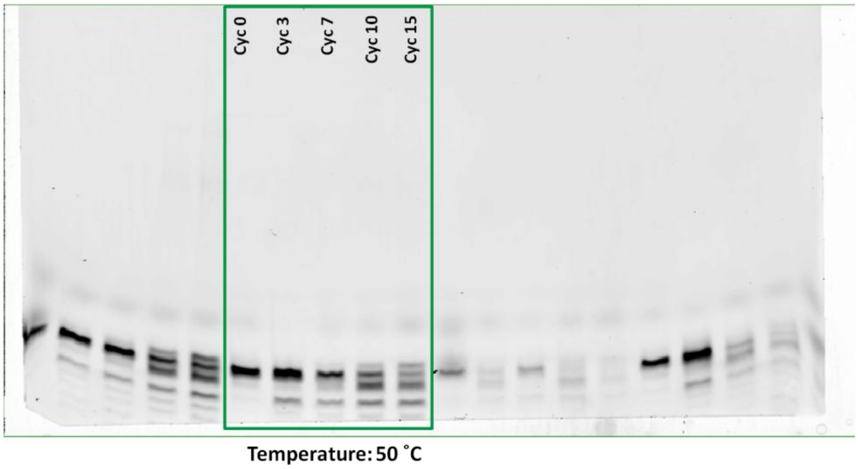

4. Figure 2a middle panel

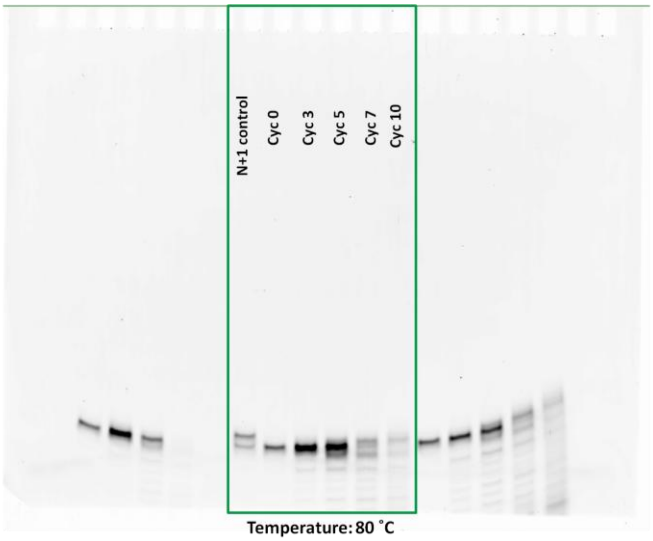

5. **Figure 2a right panel**

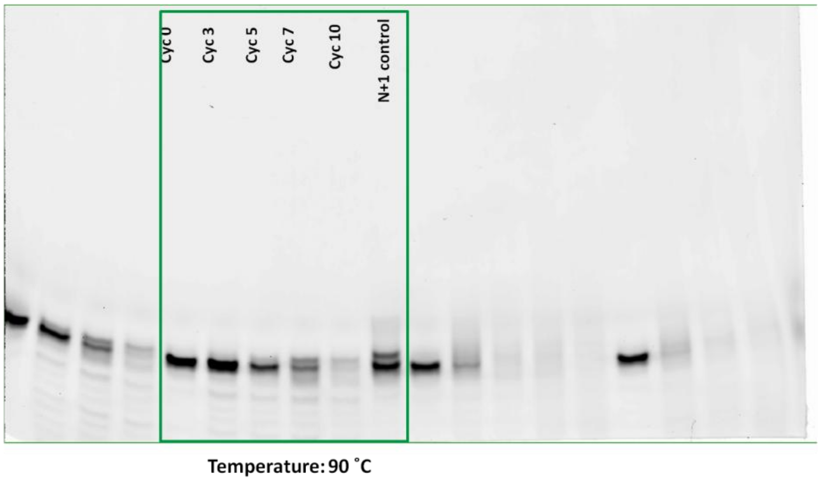

6. **Figure 2b**

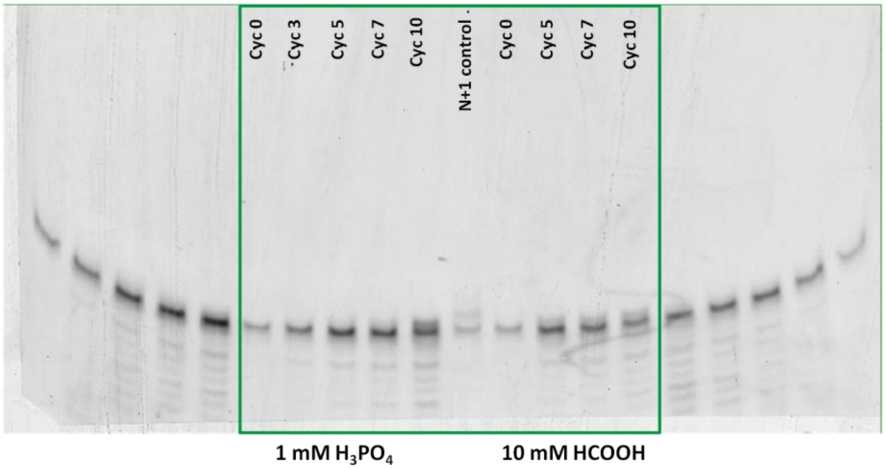

7. **Figure 3a left panel**

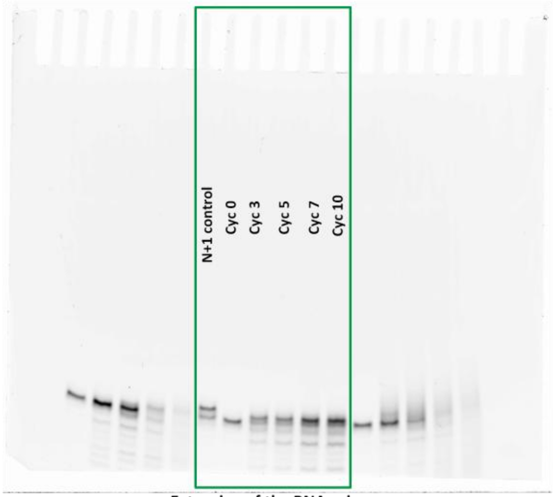

Extension of the RNA primer
using 1mM 5'-AMP and 5mM lipid

8. **Figure 3a right panel**

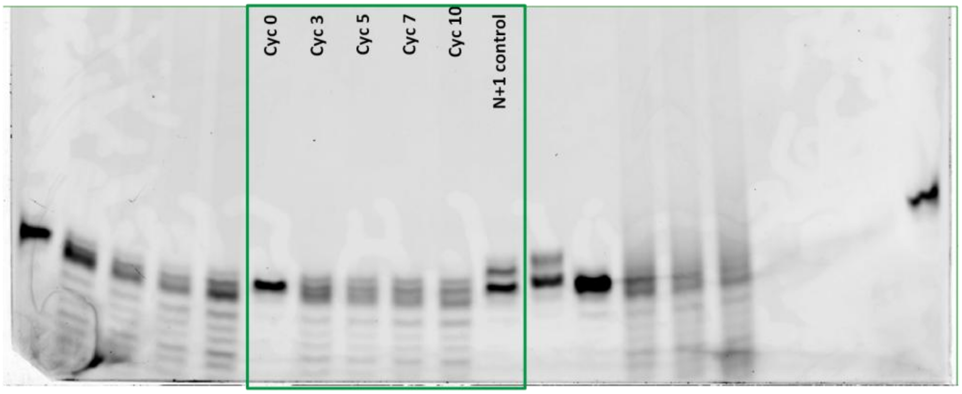

Extension of the RNA primer
using 1mM 5'-AMP and 1mM lipid

9. **Figure 3b**

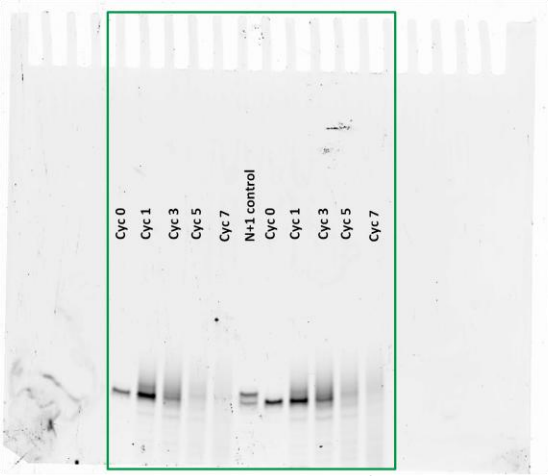

Extension of RNA primer using 5'-rMP in absence of template

Extension of RNA primer using 5'-rMP in presence of template

<u>**Un-cropped original images for pertinent figures from the supplementary information:**</u>

10. **Figure S1**

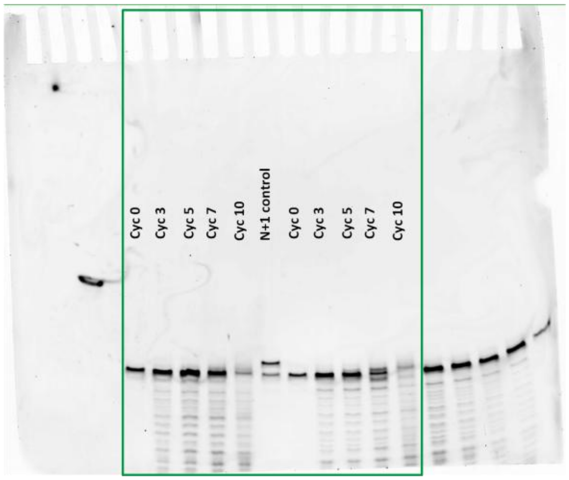

Extension of Hydroxyl G primer using MisInc_U template and 5'-AMP

Extension of Amino G primer using MisInc_U template and 5'-AMP

11. Figure S2 upper panel

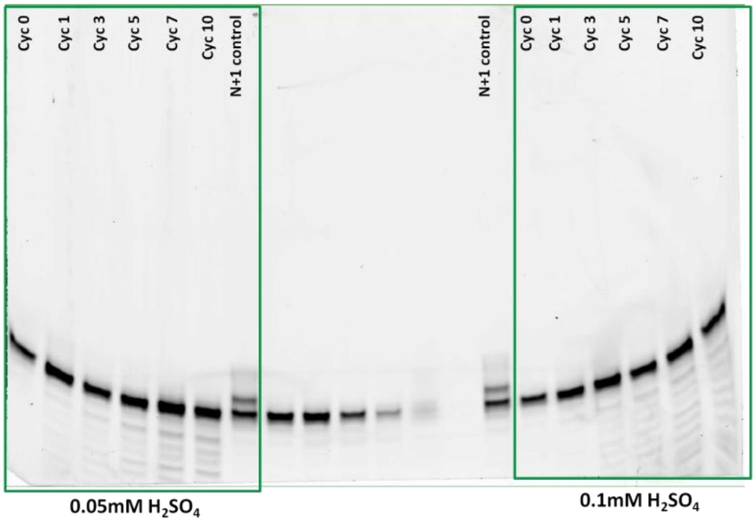

12. Figure S2 lower panel

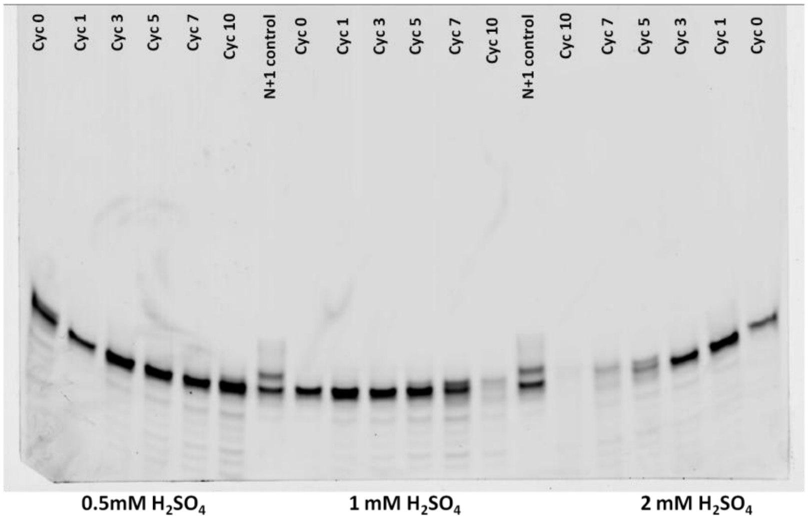

13. Figure S3

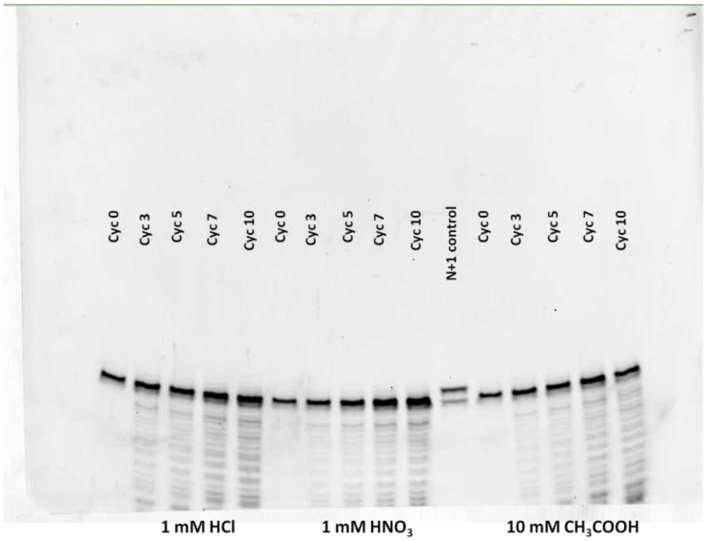

14. Figure S4

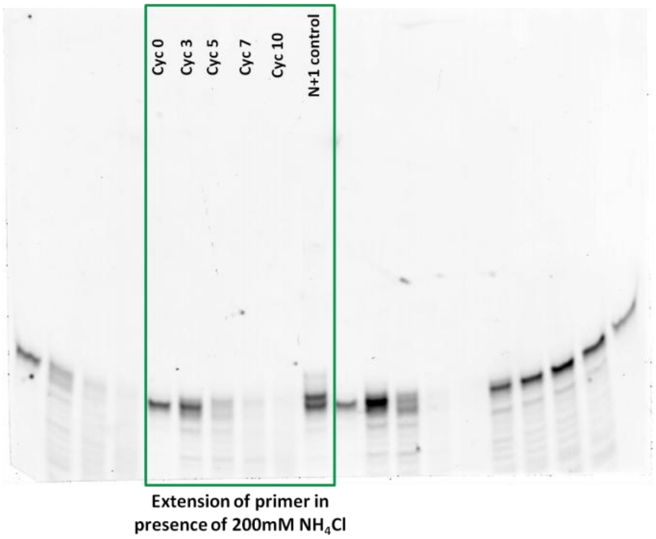

15. Figure S5

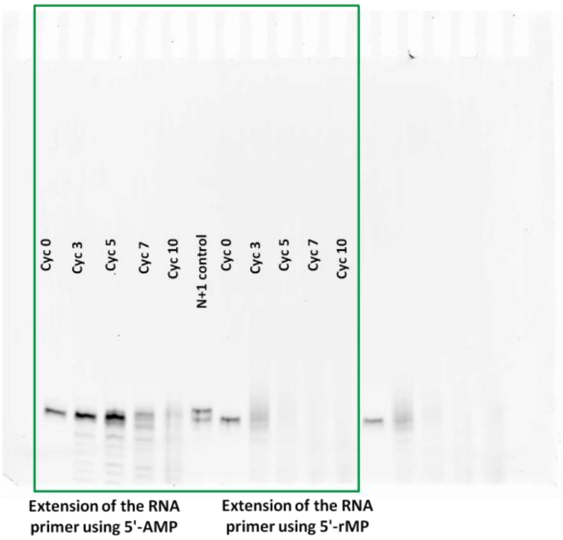

16. Figure S6

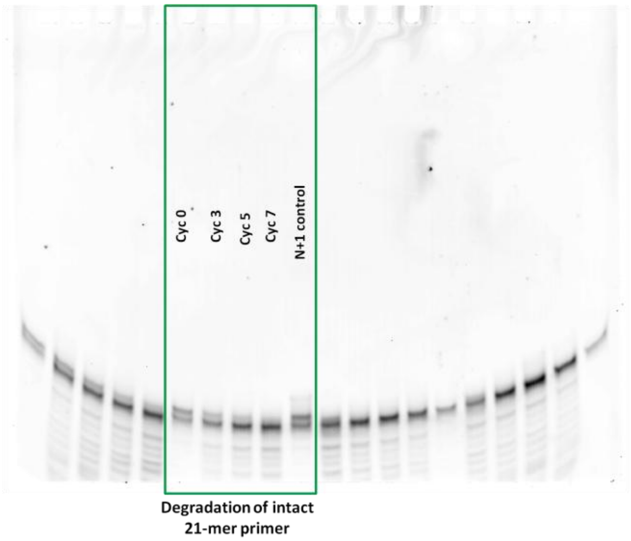

Reference:

1. Bapat, N.V., Rajamani, S. Effect of co-solutes on template-directed nonenzymatic replication of nucleic acids. J. Mol. Evol. **81**, 72-80, 10.1007/s00239-015-9700-1 (2015).
